# 16S rRNA sequencing and conventional culture provide complementary information in hospitalized patients with chronic lower-limb wounds

**DOI:** 10.3389/fcimb.2026.1865385

**Published:** 2026-08-18

**Authors:** Bartosz Molasy, Jarosław Rachuna, Monika Wawszczak-Kasza, Julia Dulębska, Katarzyna Kuszewska, Wioletta Adamus-Białek

**Affiliations:** 1 Institute of Medical Sciences, Jan Kochanowski University, Kielce, Poland; 2 Department of General Surgery and Coloproctology, St. Alexander Hospital, Kielce, Poland; 3 Diagnostic Laboratory, St. Alexander Hospital, Kielce, Poland

**Keywords:** 16S rRNA sequencing, chronic wounds, conventional culture, length of stay, wound healing, wound microbiome

## Abstract

Chronic lower-limb wounds are polymicrobial and difficult to characterize using conventional culture alone. We compared baseline wound microbiota assessed by 16S rRNA sequencing and conventional culture in 62 hospitalized adults with chronic lower-limb wounds and examined associations with subsequent length of stay. Sequencing detected a mean of 11.62 taxa per sample versus 2.24 by culture, with an overall concordance of 51.35%. In sequencing-based analyses, alpha diversity was not associated with length of stay, whereas beta diversity differed significantly according to hospitalization duration (PERMANOVA, R² = 0.030, p = 0.005), and six genera were associated with length of stay. Conventional culture showed no significant association with length of stay. No significant associations were found between sequencing-derived baseline microbiota and wound thermal parameters or 12-week healing. These findings indicate that 16S rRNA sequencing and conventional culture provide distinct yet complementary views of chronic wound microbiology.

## 1 Introduction

Chronic wounds of the lower limbs are a major challenge for healthcare due to recurrent infections, reduced mobility, lower quality of life, and frequent hospitalizations (Frykberg and Banks, 2015; Martinengo et al., 2019; Sen, 2021; Sen, 2023). Although diabetic, venous, ischemic, and mixed ulcers differ in their causes, they share several underlying pathophysiological mechanisms that contribute to chronicity, including microcirculatory impairment, tissue hypoxia, persistent inflammation, extracellular matrix dysregulation, and the presence of microbes in the wound bed (Martinengo et al., 2019; Zielińska et al., 2023; Yang et al., 2024).

In recent years, the wound microbiome has become a key aspect of understanding the biology of chronic wounds. Increasing evidence indicates that wound healing depends not only on individual microorganisms but also on the broader ecological makeup of their community, including polymicrobial interactions, anaerobic components, biofilm formation, and host–microbe communication (Oates et al., 2012; Malone et al., 2017a; Kalan et al., 2019; Verbanic et al., 2020; Molasy and Wrzosek, 2026a). Sequencing studies show that chronic wounds often harbor complex bacterial communities that differ from nearby skin and may impact healing, infection severity, and treatment effectiveness (Kalan et al., 2019; Verbanic et al., 2020; Mudrik-Zohar et al., 2022; Kunimitsu et al., 2023). At the same time, microbial signatures found in different studies are inconsistent, probably due to variations in the technical procedures and individual patients’ features (Travis et al., 2020; Verbanic et al., 2020; Mudrik-Zohar et al., 2022; Molasy and Wrzosek, 2026a).

Conventional culture is routinely used in the microbiological assessment of chronic wounds, particularly when wound infection is suspected clinically, to identify cultivable organisms and guide antimicrobial susceptibility-based treatment (Molasy and Wrzosek, 2026a). The matter becomes even more complicated in polymicrobial lesions (Oates et al., 2012; Schaper et al., 2024). 16S rRNA next-generation sequencing (NGS) provides more detailed taxonomic information at the community level and should be seen not as a substitute but as an addition to traditional microbiology, providing better insights into complex wound ecosystems (Oates et al., 2012; Malone et al., 2017b; Mudrik-Zohar et al., 2022).

This is particularly relevant in hospitalized patients with chronic wounds, in whom longer hospital stay usually reflects greater disease burden, more complex treatment, and higher use of healthcare resources (Martinengo et al., 2019; Malone and Schultz, 2022; Sen, 2023). In our recent review, we summarized the rationale for microbiome-informed wound assessment and discussed the potential role of the wound microbiome as both a biomarker and a therapeutic target (Molasy and Wrzosek, 2026a). Additionally, our recent work on chronic lower-limb ulcers showed that thermographic and inflammatory variables may support early risk stratification in this patient group (Molasy and Wrzosek, 2026b). Building on this research, the current study aims to profile baseline wound microbiota through 16S rRNA NGS, compare these findings with traditional culture methods, and determine if initial microbial patterns correlate with length of stay in patients with chronic lower-limb wounds. Additional analyses examined microbiome associations with wound thermal parameters and 12-week healing.

## 2 Methods

### 2.1 Study design and participants

This single-center observational study was conducted between January and September 2025 in a surgical center specializing in the management of chronic wounds. Adult patients referred for inpatient treatment of chronic lower-limb wounds were screened for eligibility. The study population included patients hospitalized for chronic lower-limb wounds requiring inpatient treatment, frequently in the setting of suspected wound infection. However, the presence of clinical signs of infection was not applied as a formal inclusion or exclusion criterion. Inclusion criteria were age >18 years and a chronic, non-healing or difficult-to-heal wound persisting for more than six weeks. Exclusion criteria were wound area >300 cm², lack of informed consent, absence of an identified underlying disease impairing wound healing, and critical limb ischemia. Wounds >300 cm² were excluded because very extensive lesions could not be reliably assessed using the standardized thermographic protocol applied in this study and would also introduce substantial local heterogeneity in region-of-interest analysis.

Baseline demographic and clinical data were collected from medical history and physical examination and included wound etiology, comorbidities, previous treatment, routine laboratory parameters, and infrared thermography. Samples were collected at baseline, at the time of hospital admission, after initial clinical evaluation. For microbiological assessment, after a surgical debridement, a wound swab was collected for conventional culture, and a wound tissue specimen was collected for 16S rRNA sequencing-based microbiome profiling. Patients were subsequently treated according to routine clinical practice and followed for predefined short-term clinical outcomes. The protocol followed the Declaration of Helsinki and received approval from the Institutional Bioethics Committee (approval no. 4/2025). All participants provided written informed consent to participate in the study. The study followed the STROBE reporting guidelines for observational research.

### 2.2 Clinical data collection and outcomes

Baseline variables included age, sex, wound etiology, comorbidities, treatment history, and routine laboratory parameters. Wound assessment included clinical evaluation and infrared thermographic measurements. The primary outcome was length of stay. Secondary outcomes were wound temperature, temperature gradient (ΔT), healing within 12 weeks, and need for amputation. Complete wound healing was defined as full epithelial closure without drainage, as determined by clinical assessment at 12 weeks.

### 2.3 Thermographic assessment

At baseline, all participants underwent infrared thermographic assessment according to a standardized protocol using the same device (FLIR One Pro; Teledyne FLIR LLC, Wilsonville, OR, USA). All measurements were performed at baseline before outcome data were available. Thermographic assessment was performed by a single trained investigator, minimizing interoperator variability. Images were analyzed immediately at the bedside, and values were recorded prospectively in the study database.

Examinations took place in a thermally stable environment. The room temperature was kept at 22 ± 1 °C, and the relative humidity was about 50%. No directed airflow was allowed toward the limb during imaging. Before we started, patients acclimatized for 10 minutes to minimize artefacts from recent changes in limb position or temperature. We removed the dressings shortly before imaging and left the wound uncovered for 60 to 90 seconds prior to acquisition to minimize temporary temperature artifacts. The camera was positioned at a fixed distance (30 cm) and perpendicular to the wound surface.

The wound bed and a periwound zone beyond the wound margin were analyzed as regions of interest. Absolute wound-bed temperature and the wound-to-periwound temperature gradient (ΔT = T_bed − T_peri) were recorded for further analyses. Two manually defined regions of interest were used: the wound bed and a periwound zone extending 1–2 cm from the wound margin. To improve consistency, the coldest identifiable point within the wound bed was used to represent wound-bed temperature, whereas the warmest point within macroscopically intact surrounding skin located approximately 1–2 cm beyond the wound edge was used to represent periwound temperature. This pragmatic approach was chosen to capture the most compromised wound area and the most inflamed periwound zone while preserving bedside feasibility.

### 2.4 Conventional microbiology

Wound swabs were obtained using the Levine technique after debridement of the ulcer bed and cultured in accordance with standard clinical practice. Species identification and antimicrobial susceptibility testing were performed using the Phoenix M50 analyzer (Becton Dickinson), with interpretation according to current EUCAST recommendations. For the present analysis, culture results were further categorized according to microbial diversity and taxonomic composition for comparison with 16S rRNA sequencing findings.

### 2.5 DNA isolation, library preparation and 16S rRNA sequencing

Wound tissue specimens were frozen at −80 °C immediately after collection until further processing. Approximately 30 mg of tissue was used for downstream extraction. Before DNA isolation, tissues were homogenized using TissueLyser III (Qiagen), and microbial DNA was extracted using the QIAamp Fast DNA Tissue Kit (Qiagen) according to the manufacturer’s protocol, including mechanical bead-beating. In the extended protocol, DNA was eluted in Buffer ATE and stored at −80 °C. DNA quantity and purity were assessed fluorometrically using Quantus (Promega) with the QuantiFluor ONE dsDNA system.

Libraries targeting the V3–V4 regions of the bacterial 16S rRNA gene were prepared using the Illumina 16S Metagenomic Sequencing Library Preparation protocol. DNA samples were normalized to 5 ng/μL before PCR amplification of the V3–V4 hypervariable regions. Amplicons were indexed using the Nextera XT Index Kit v2 Set A (Illumina), purified with MagSi-NGS PREP Plus beads, and quality-checked by automated electrophoresis using the TapeStation platform with D1000 ScreenTape reagents. Pooled libraries were sequenced on the Illumina MiSeq DX platform using paired-end reads (2 × 300 bp; MiSeq Reagent Kit v3) with 15% PhiX spike-in to increase sequence diversity.

### 2.6 Bioinformatic processing

Raw paired-end reads were quality-filtered and processed in QIIME 2. Preprocessing included Trim Galore! (version 0.6.4) to remove adapter sequences and low-quality reads, followed by import into QIIME 2 using the PairedEndFastqManifestPhred33V2 format. Amplicon sequence variants were inferred with DADA2. 24 base pairs were trimmed from the 5′ ends of both forward and reverse reads, and reads were truncated to 260 bp for forward reads and 250 bp for reverse reads. Taxonomic assignment was performed against the SILVA 138 reference database using the VSEARCH consensus classifier with a 97% identity threshold.

### 2.7 Statistical analysis

Microbiome analyses were conducted in R using phyloseq and related packages. Data processing and visualization were performed in R using phyloseq and ggplot2. Sequencing depth was high, with a median of 68,351 reads per sample and a mean of 67,267 reads per sample. Rarefaction analysis indicated taxonomic saturation at approximately 5,000 reads per sample. To standardize library size for diversity analyses, samples were rarefied to a uniform depth; one sample with insufficient coverage was excluded after rarefaction.

Alpha diversity was evaluated using the Shannon index and compared with non-parametric tests. Wilcoxon rank-sum and Kruskal–Wallis tests were used as appropriate. Continuous variables used for diversity comparisons were categorized using prespecified thresholds or median-based cut-offs. Beta diversity was calculated using Bray–Curtis distance matrices, visualized by principal coordinates analysis, and tested using permutational multivariate analysis of variance (PERMANOVA) with 999 permutations.

Phylum-level taxonomic composition was summarized as relative abundance after taxonomic agglomeration. In comparative analyses with conventional microbiology, taxa with a relative abundance below 10% within a sample were excluded to reduce the impact of very low-abundance signals of uncertain clinical relevance. Differential abundance analysis was performed with DESeq2 after conversion from the phyloseq format, using a negative binomial model and false discovery rate correction according to the Benjamini–Hochberg procedure. For descriptive comparison between methods, full agreement referred to concordant microbiological findings between conventional culture and sequencing at the sample level according to the predefined comparison framework. Dispersion estimates were obtained using the poscounts method. For analyses involving continuous clinical variables, effect estimates were expressed as log2 fold-change per additional hospital day or per degree of temperature change. Adjusted p-values <0.05 were considered statistically significant.

## 3 Results

### 3.1 Baseline characteristics of the study group

A total of 62 patients with chronic lower-limb wounds were included in the study. The mean age was 68 years, and 38 patients (61.3%) were male. The cohort included wounds of mixed etiology, most commonly venous (27.4%) and diabetic (25.8%), followed by ischemic (14.5%), mixed (12.9%), post-traumatic (11.3%), and pressure ulcers (8.1%).

Mean wound-bed temperature was 30.18 °C, mean wound-to-periwound temperature gradient (ΔT) was −2.74 °C, and mean length of stay was 9.77 days. Complete wound healing within 12 weeks was observed in 23 patients (37.1%), whereas 25 patients (40.3%) underwent amputation during treatment. Baseline demographic and clinical characteristics are summarized in **Table 1**.

**Table 1:** Baseline characteristics of the study cohort.

Variable	Value
Patients, n	62
Age, years	68
Male sex, n (%)	38 (61.3%)
Obesity, n (%)	28 (45.2%)
Diabetes, n (%)	40 (64.5%)
Current smoking, n (%)	29 (46.8%)
Previous vascular intervention, n (%)	6 (9.7%)
Previous amputation, n (%)	23 (37.1%)
Wound duration, weeks	22.34
Wound etiology, n (%)	
– Venous	17 (27.4%)
– Diabetic	16 (25.8%)
– Ischemic	9 (14.5%)
– Post-traumatic	7 (11.3%)
– Mixed	8 (12.9%)
– Pressure injury	5 (8.1%)
Wound-bed temperature, °C	30.18
ΔT, °C	-2.74
Length of stay, days	9.77
Healed within 12 weeks, n (%)	23 (37.1%)
Amputation during treatment, n (%)	25 (40.3%)

Continuous variables are presented as mean, and categorical variables as number (%). ΔT was calculated as wound-bed temperature minus periwound temperature.

### 3.2 Baseline comparison of conventional culture and 16S rRNA sequencing

Compared with conventional culture, 16S rRNA sequencing revealed substantially broader baseline microbial diversity. The mean number of detected taxa per sample was 11.62 with sequencing and 2.24 with conventional culture, corresponding to a mean detection surplus of 9.38 taxa per sample and a median increase in detection sensitivity of 550%. Full agreement between methods was observed in 19 samples, whereas 18 samples showed discrepant findings. Conventional microbiology was negative in 26 cases. Overall concordance between methods was 51.35% (**Table 2**).

**Table 2:** Comparison of baseline microbiological findings obtained by 16S rRNA sequencing (NGS) and conventional culture.

Parameter/metric	NGS	Culture-based method
Quantitative characteristics		
Mean number of identified taxa	11.62	2.24
Median number of identified taxa	12	2
Detection efficiency comparison	Value	Unit
Mean detection surplus (NGS vs. Culture)	9.38	taxa/sample
Median detection surplus	10	taxa/sample
Mean increase in detection sensitivity (NGS)	586.94	%
Median increase in detection sensitivity (NGS)	550	%
Methodological agreement		
Full agreement samples (NGS = Culture)	19	n
Discrepant samples (NGS ≠ Culture)	18	n
Overall Concordance Rate	51.35	%
Negative samples in traditional microbiology	26	n

The table summarizes the number of detected taxa, the detection surplus of 16S rRNA sequencing relative to conventional culture, and the agreement between methods at baseline. Negative samples refer to cases with no growth in conventional microbiology.

### 3.3 Baseline microbiological findings and subsequent length of stay

**Figure 1:**
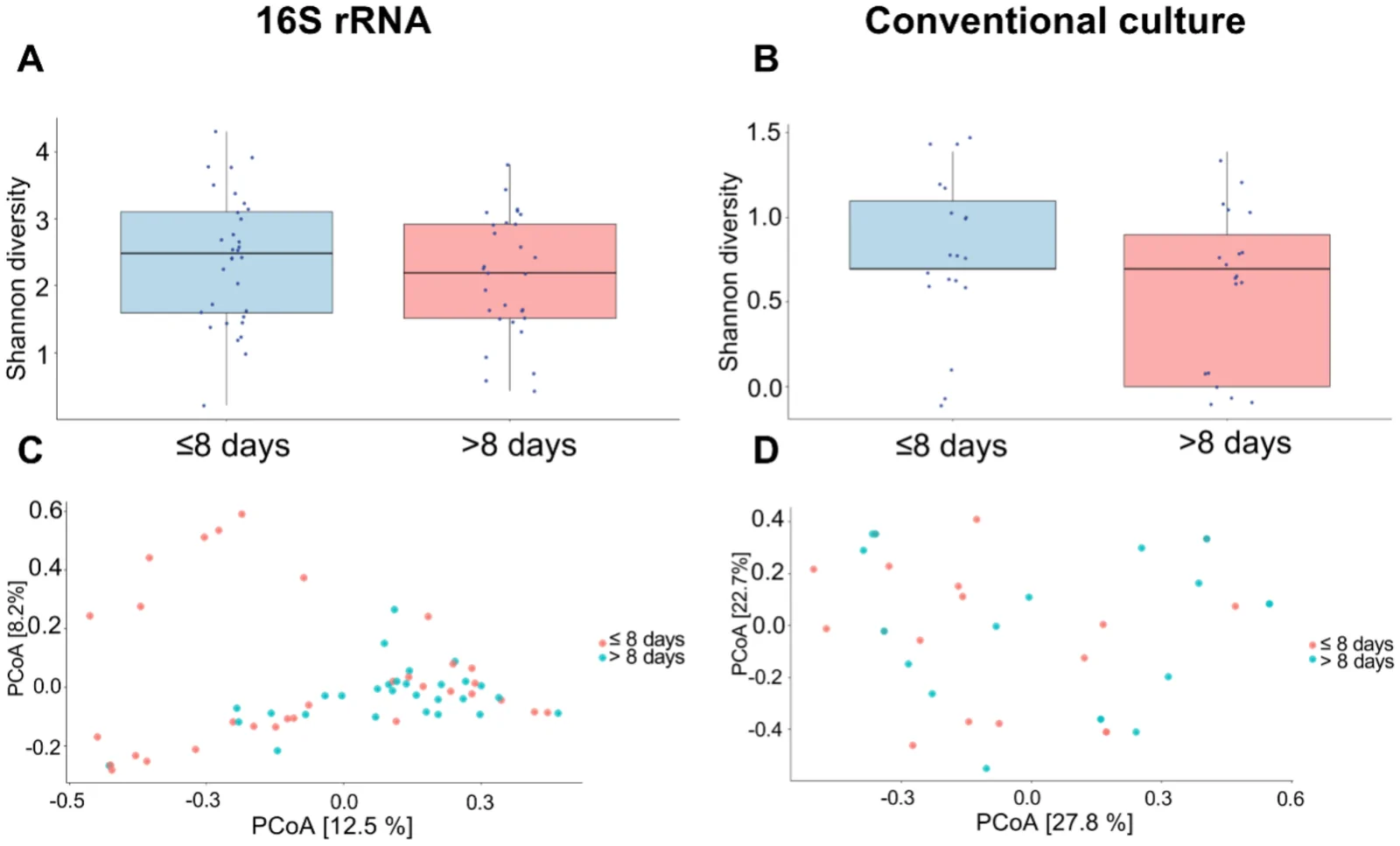
Baseline wound microbiota and subsequent length of stay in 16S rRNA sequencing and conventional culture analyses. **(A)** Alpha diversity (Shannon index) in NGS-based analysis according to length of stay (≤8 vs. >8 days). **(B)** Alpha diversity (Shannon index) in conventional culture-based analysis according to length of stay (≤8 vs. >8 days). **(C)** Principal coordinates analysis (PCoA) based on Bray–Curtis distances in NGS-based analysis. **(D)** Principal coordinates analysis (PCoA) based on Bray–Curtis distances in conventional culture-based analysis.

In NGS-based analyses, we did not find a significant difference in alpha diversity between patients hospitalized for 8 days or less and those hospitalized for more than 8 days (Shannon index, p = 0.385; **Figure 1A**). However, beta diversity differed significantly with hospitalization length (PERMANOVA, R² = 0.030, p = 0.005; **Figure 1C**). These findings indicate that baseline community composition was associated with length of stay, although the effect size was small. In conventional culture-based analyses, we found no significant connection with hospitalization length for either alpha diversity (p = 0.180; **Figure 1B**) or beta diversity (PERMANOVA R² = 0.033, p = 0.313; **Figure 1D**).

**Figure 2:**
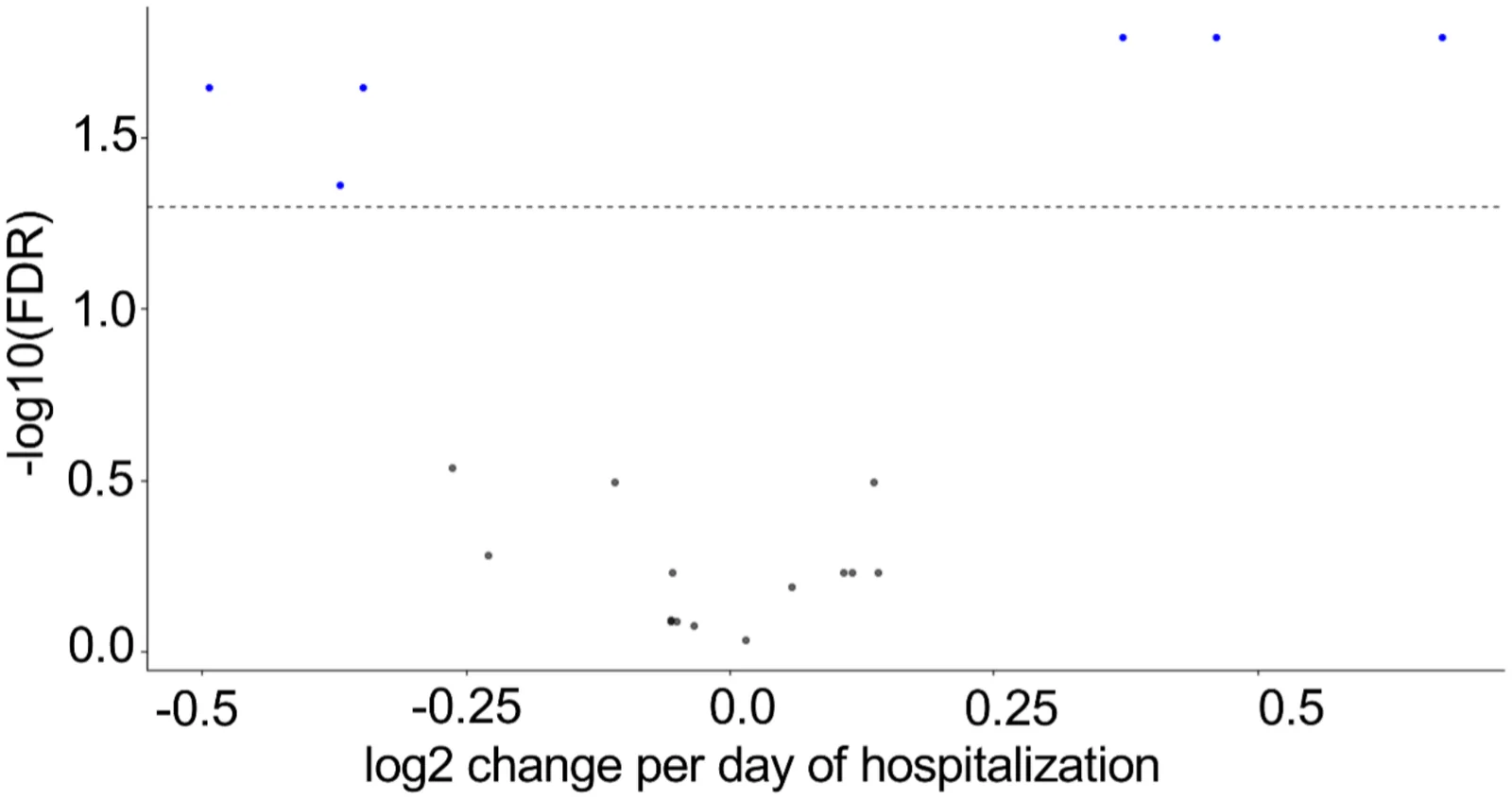
Differential abundance analysis of baseline wound microbiota in relation to subsequent length of hospital stay. Volcano plot illustrating differential abundance per day of hospitalization. Each point represents a single taxon. The dashed horizontal line indicates the significance threshold (FDR = 0.05). Blue points represent taxa with statistically significant changes, while grey points denote non-significant findings.

Differential abundance analysis identified six genera associated with length of stay (**Table 3**, **Figure 2**). *Corynebacterium*, *Dialister*, and *Proteus* were associated with each additional hospital day, while *Morganella*, *Paracoccus*, and *Enhydrobacter* had the opposite effect. *Corynebacterium* had the strongest positive association (log2FC 0.676, adjusted p = 0.016). In contrast, *Morganella* showed the strongest negative association (log2FC −0.494, adjusted p = 0.023).

**Table 3:** Genera associated with subsequent length of stay in differential abundance analysis of 16S rRNA sequencing data.

Phylum	Genus	log2FC per hospital day	SD	Adjusted *p*-value (FDR)
*Actinobacteriota*	*Corynebacterium*	0.676	0.205	0.016
*Firmicutes*	*Dialister*	0.460	0.148	0.016
*Proteobacteria*	*Proteus*	0.373	0.123	0.016
*Proteobacteria*	*Morganella*	-0.494	0.176	0.023
*Proteobacteria*	*Paracoccus*	-0.369	0.149	0.044
*Proteobacteria*	*Enhydrobacter*	-0.347	0.125	0.023

log2FC values represent the change in relative abundance per additional hospital day. Positive values indicate genera associated with longer hospitalization, whereas negative values indicate inverse associations. Adjusted *p*-values were calculated using the Benjamini–Hochberg false discovery rate procedure.

At the phylum level, taxonomic composition was broadly similar between hospitalization groups. *Proteobacteria*, *Firmicutes*, and *Bacteroidota* were dominant in both groups, with only modest shifts in relative abundance and no major restructuring of the overall phylum-level profile (**Supplementary Figure 1**).

### 3.4 Baseline microbiota in relation to intrawound temperature

**Figure 3:**
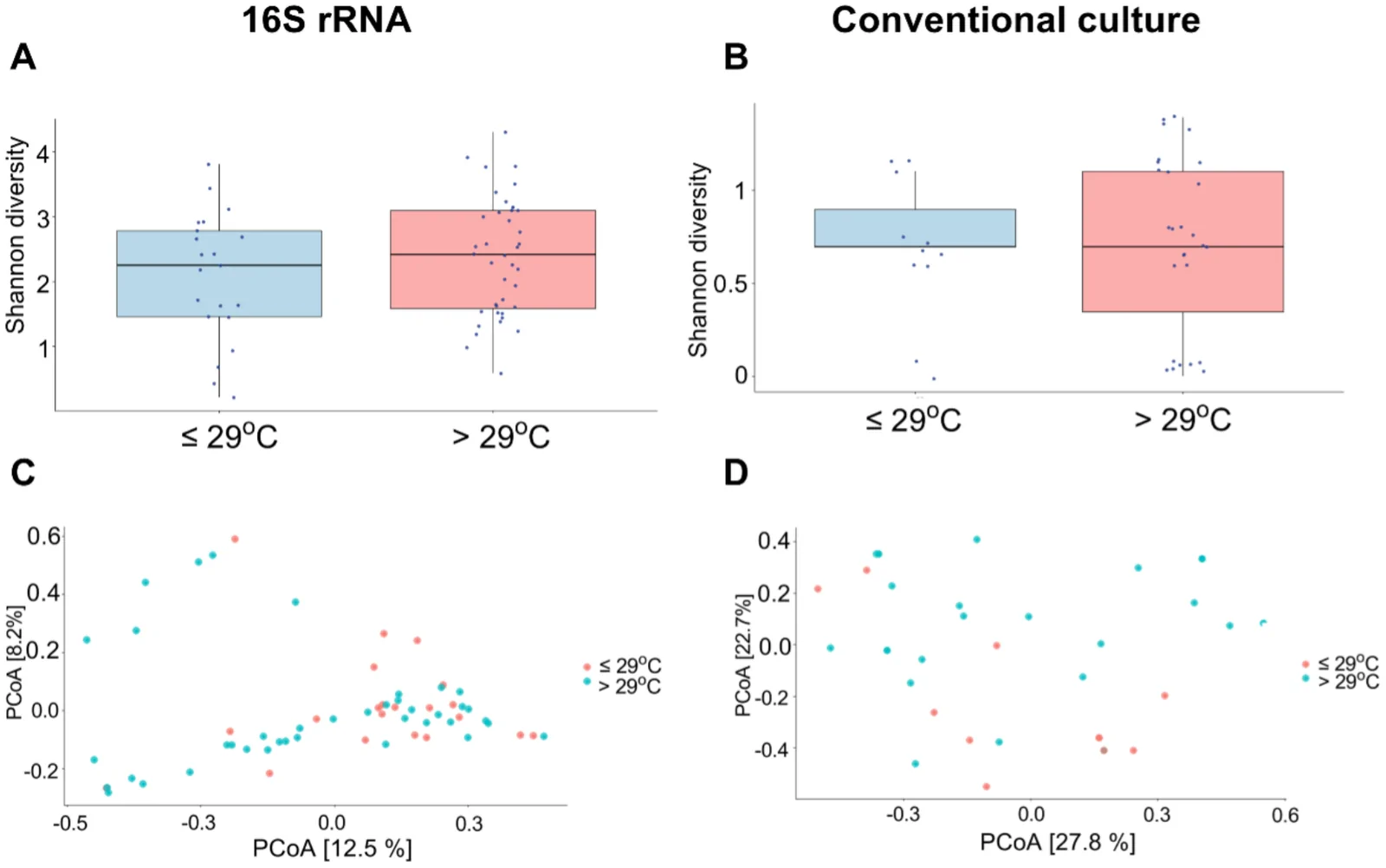
Baseline wound microbiota in relation to intrawound temperature in 16S rRNA sequencing and conventional culture analyses. **(A)** Alpha diversity (Shannon index) in NGS-based analysis according to intrawound temperature category (≤29 °C vs. >29 °C). **(B)** Alpha diversity (Shannon index) in conventional culture-based analysis according to intrawound temperature category (≤29 °C vs. ≥ 29 °C). **(C)** Principal coordinates analysis (PCoA) based on Bray–Curtis distances in NGS-based analysis. **(D)** Principal coordinates analysis (PCoA) based on Bray–Curtis distances in conventional culture-based analysis.

In sequencing-based analyses, baseline microbiota showed no significant association with intrawound temperature for alpha diversity, beta diversity, or differential abundance (**Figures 3A,C**). In conventional culture-based analyses, alpha diversity also did not significantly relate to intrawound temperature (p = 0.760; **Figure 3B**). However, beta diversity varied significantly between temperature groups (PERMANOVA R² = 0.080, p = 0.010; **Figure 3D**). No significant genera were found in the differential abundance analysis for this endpoint. At the phylum level, *Proteobacteria*, *Firmicutes*, and *Bacteroidota* remained dominant across temperature categories, showing no major changes in the overall profile (**Supplementary Figure 2**).

### 3.5 Baseline microbiota in relation to wound-to-periwound temperature gradient

No significant links were found between baseline microbiota and the wound-to-periwound temperature gradient (ΔT) in either sequencing-based or culture-based analyses. This included analyses of alpha and beta diversity and differential abundance. The phylum-level composition also stayed consistent across ΔT categories, with *Proteobacteria*, *Firmicutes*, and *Bacteroidota* being the main phyla in both groups (**Supplementary Figure 3**).

### 3.6 Baseline microbiota in relation to 12-week healing

**Figure 4:**
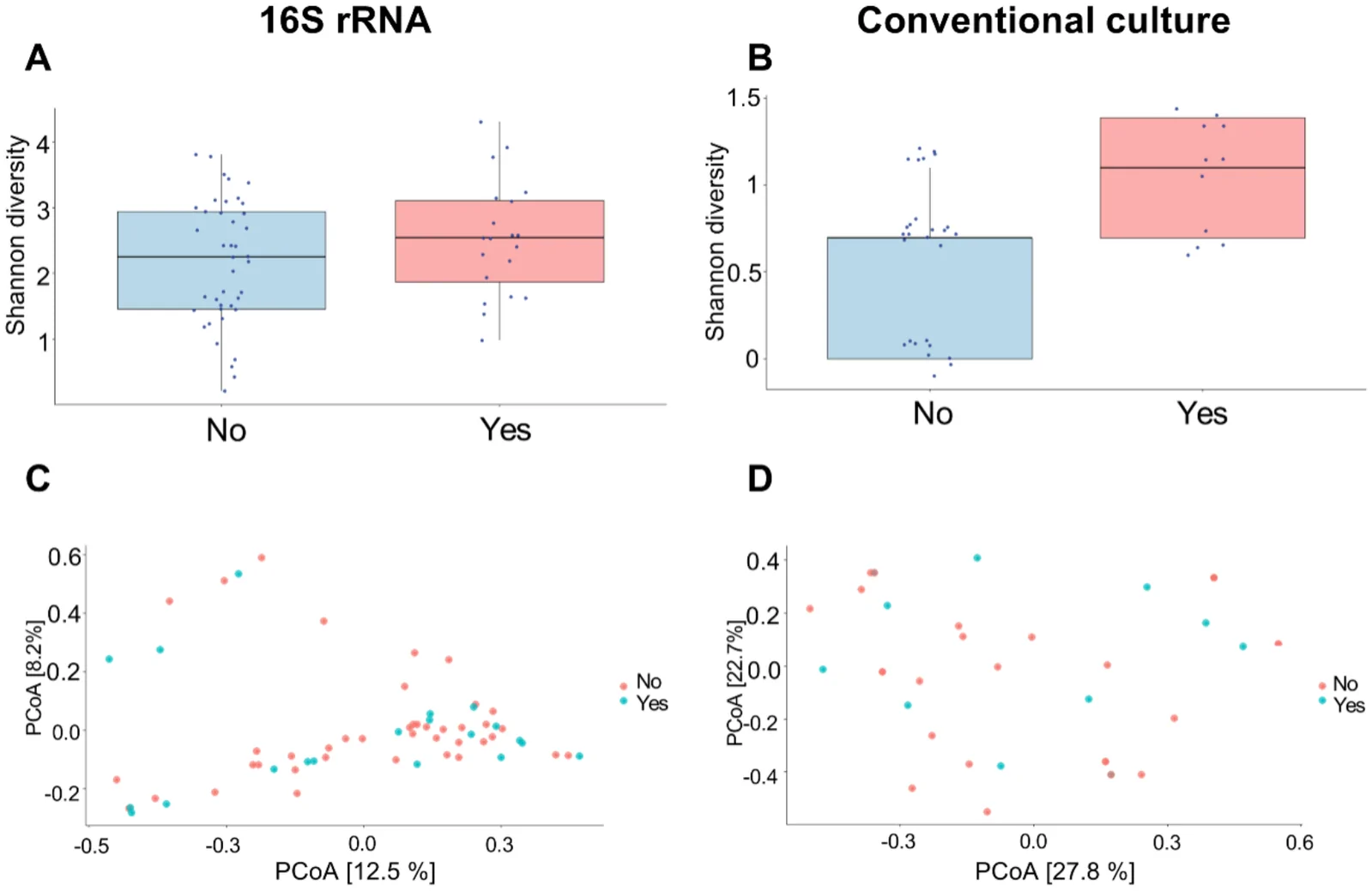
Baseline wound microbiota in relation to 12-week healing in 16S rRNA sequencing and conventional culture analyses. **(A)** Alpha diversity (Shannon index) in NGS-based analysis according to 12-week healing status. **(B)** Alpha diversity (Shannon index) in conventional culture-based analysis according to 12-week healing status. **(C)** Principal coordinates analysis (PCoA) based on Bray–Curtis distances in NGS-based analysis. **(D)** Principal coordinates analysis (PCoA) based on Bray–Curtis distances in conventional culture-based analysis.

In sequencing-based analyses, we did not find any significant associations between baseline microbiota and 12-week healing status for alpha diversity, beta diversity, or differential abundance (**Figures 4A,C**). In conventional culture-based analyses, the 12-week healing period was significantly associated with alpha diversity (p = 0.002; **Figure 4B**). However, no significant association was found for beta diversity (**Figure 4D**). We did not identify any significant differences in taxa between healing status groups. The composition at the phylum level was largely the same for patients with and without wound healing within 12 weeks. *Proteobacteria*, *Firmicutes*, and *Bacteroidota* remained the dominant phyla, with no major changes in the overall phylum-level structure (**Supplementary Figure 4**).

## 4 Discussion

The main finding of this study is that the initial wound microbiota at hospital admission was associated with the length of stay. The strongest relationship was with community composition, not overall alpha diversity. At the same time, standard culture methods and 16S rRNA sequencing did not show the same clinical connections. Sequencing-based analyses correlated with the length of stay, while culture-based methods indicated links with intrawound temperature and healing after 12 weeks. Taken together, these findings suggest that the two methods capture complementary aspects of chronic wound microbiology.

The link between initial microbiota and length of stay is biologically plausible. In patients with chronic lower-limb wounds, longer hospital stays usually result from factors such as local infection, tissue damage, repeated debridement, systemic inflammation, limb-salvage complexity, and other coexisting comorbidities (Frykberg and Banks, 2015; Malone and Schultz, 2022; Popescu et al., 2023; Sen, 2023). Baseline microbial composition may reflect a wound environment that is more difficult to control during hospitalization. Previous research indicates that the microbiota in chronic wounds and diabetic foot ulcers can relate to clinical outcomes, healing patterns, and complications, although the exact microbial signatures reported across cohorts have varied significantly (Smith et al., 2016; Malone et al., 2017b; Suryaletha et al., 2018; Kalan et al., 2019; Park et al., 2019; Travis et al., 2020; Verbanic et al., 2020; Mudrik-Zohar et al., 2022; Kunimitsu et al., 2023; Wang et al., 2023). Our findings build on this idea by suggesting that, in a mixed-etiology group of hospitalized patients, the most noticeable signal in the baseline microbiome may reflect the burden of short-term outcomes rather than the eventual healing of wounds.

The signal related to length of stay in our study was mainly driven by beta diversity and differential abundance analyses, rather than by a significant difference in Shannon diversity. This suggests that the clinically relevant signal was related more to community structure and specific taxa than to overall richness or evenness. Such a pattern aligns with current understanding of chronic wound microbiology. In this field, the organization of multiple species, ecological interactions, and the selective enrichment of certain genera may provide more information than diversity indices alone (Oates et al., 2012; Smith et al., 2016; Malone et al., 2017b; Verbanic et al., 2020; Mudrik-Zohar et al., 2022; Molasy and Wrzosek, 2026a). Taxon-level findings should still be interpreted with caution. Among genera positively associated with longer hospitalization, *Corynebacterium*, *Dialister*, and *Proteus* might reflect a wound environment with greater microbiological complexity or persistence, whereas *Morganella*, *Paracoccus*, and *Enhydrobacter* showed inverse associations. Of these, *Corynebacterium* is particularly noteworthy, as some species have been described as clinically relevant opportunists in diabetic foot ulcers and in hospitalized patients (Zheng et al., 2024). *Proteus* is also plausible in this context, given its well-known role in chronic wound colonization and persistence (Hall-Stoodley et al., 2004; Oates et al., 2012). However, these findings should be seen as associative and hypothesis-generating rather than definitive prognostic markers.

The present findings can also be interpreted in the context of chronic wound biofilms. Biofilm-associated communities are thought to contribute to persistence, impaired healing, and reduced responsiveness to treatment by promoting microbial tolerance, chronic inflammation, and complex interspecies interactions (Uberoi et al., 2024; Shen et al., 2025). Although our study did not directly assess biofilm architecture, the observed association of community composition with length of stay is consistent with the broader concept that polymicrobial wound organization, rather than the presence of individual taxa alone, may be clinically relevant.

A key practical aspect of this study is the parallel assessment of conventional culture and NGS. Our results support their complementary roles, rather than suggesting that one method is better than the other. Conventional culture is crucial for identifying culturable organisms that have direct therapeutic implications and for guiding antimicrobial treatment (Schaper et al., 2024). On the other hand, NGS provides a broader ecological perspective, including polymicrobial communities and taxa that may be underrepresented in routine culture, particularly anaerobic or low-abundance organisms (Malone et al., 2017b; Suryaletha et al., 2018; Huang et al., 2022; Mudrik-Zohar et al., 2022; Molasy and Wrzosek, 2026a). Although sequencing can detect low-abundance microorganisms beyond the reach of routine culture, the clinical relevance of such signals remains uncertain. Some low-abundance taxa may represent transient colonizers, contaminants, or biologically minor components of the wound ecosystem rather than actionable pathogens. For this reason, taxa below 10% relative abundance were excluded from selected comparative analyses to reduce the influence of signals with uncertain clinical interpretability.

In our dataset, NGS showed a stronger association with length of stay. Conventional culture showed specific associations with intrawound temperature and culture-based alpha diversity in relation to 12-week healing. Our results support a complementary role for both methods rather than the superiority of either approach. From a practical perspective, 16S rRNA sequencing is unlikely to replace routine culture in everyday wound diagnostics, particularly in low-resource settings, because of its cost, turnaround time, and limited availability. Its more realistic role may lie in selected complex cases, research settings, culture-negative but clinically suspicious wounds, or in studies aimed at understanding polymicrobial wound ecology beyond routine diagnostics.

The findings about wound thermal parameters are also important. In sequencing-based analyses, baseline microbiota did not show a significant link to intrawound temperature or ΔT. However, in culture-based analyses, intrawound temperature was related to beta diversity. One explanation is that local thermal conditions may be more closely related to the viable, culturable fraction of the wound microbiota than to the broader community profile identified by sequencing. This would be biologically plausible, because superficial wound temperature reflects a combination of local perfusion, inflammatory activity, and tissue compromise, all of which may influence the growth conditions of cultivable organisms (Ramirez-GarciaLuna et al., 2022; Rahman et al., 2025; Molasy and Wrzosek, 2026b). At the same time, the absence of a distinct sequence signal indicates that thermographic features and microbiome structure are connected but not interchangeable components of wound pathophysiology.

The connection between baseline microbiota and healing after 12 weeks needs careful consideration. In our study, 16S rRNA sequencing did not show a strong link between the initial community structure and healing outcomes. However, traditional culture-based alpha diversity was related to healing at 12 weeks. This may suggest that lower diversity within the culturable fraction, possibly reflecting dominance of one or a few viable taxa, is associated with poorer outcome. Additionally, healing is influenced by many factors beyond baseline microbiology. These include revascularization, offloading, quality of debridement, local wound care, exposure to antimicrobials, glycemic control, nutritional status, and the host’s immune response (Frykberg and Banks, 2015; Malone et al., 2017b; Popescu et al., 2023; Schaper et al., 2024). These competing factors may reduce the detectable impact of a single baseline microbiome snapshot. Past research connecting microbiome composition to healing frequently involved more uniform diabetic foot ulcer groups, repeated longitudinal sampling, or more detailed sequencing methods (Kalan et al., 2019; Park et al., 2019; Verbanic et al., 2020; Mudrik-Zohar et al., 2022; Wang et al., 2023). Our findings do not exclude the microbiome’s influence on wound healing. Rather, they indicate that its role might vary depending on the context and may not be fully captured by a single baseline measurement in a group of hospitalized patients with diverse causes.

The absence of strong correlations for ΔT in both sequencing-based and culture-based analyses should be highlighted. Physiologically, ΔT is a logical indicator of local temperature differences between the wound bed and surrounding skin. Yet, it did not identify microbial patterns in this group, possibly due to the complex factors influencing temperature gradients in chronic wounds. Elements such as blood flow issues, swelling, inflammation, dressing history, and anatomical variations can all impact the measured signal. Although the wound seemed to show some association in culture-based analyses, ΔT did not serve as a useful microbiological indicator in this study.

This study has several strengths. It included baseline sampling performed at hospital admission and parallel assessment using both sequencing- and culture-based microbiology. The real-world cohort consisted of hospitalized patients with chronic lower-limb wounds of mixed etiology. Integrating microbiological data with clinically relevant outcomes, including length of stay, thermographic parameters, and 12-week healing, also adds practical value. However, several limitations should be acknowledged. The sample size was modest, the cohort was biologically heterogeneous, and the observational design precludes causal inference. In addition, conventional microbiology and 16S rRNA sequencing were performed on different specimen types (swab vs tissue), which reduced direct comparability between the two methods and may have favored broader detection in tissue-based sequencing analyses. Furthermore, 16S rRNA sequencing does not provide strain-level resolution and cannot directly characterize fungal communities, resistance determinants, or virulence factors. Microbiota was assessed only at baseline, whereas chronic wound ecosystems are dynamic and may change substantially during treatment. Future studies integrating microbiome profiling with host-response markers, such as inflammatory biomarkers or cytokine patterns, may help better explain the relationship between microbial community structure and clinical outcomes (Mihai et al., 2024).

## 5 Conclusions

Baseline wound microbiota, as measured by 16S rRNA sequencing, is associated with length of stay. In contrast, conventional culture shows specific connections with intrawound temperature and healing at 12 weeks. Rather than representing competing approaches, conventional culture and sequencing appear to provide complementary information on chronic wound microbiology. Larger studies employing standardized sampling and long-term designs are necessary. These should directly compare various microbiological methods to determine the most effective approach for incorporating sequencing-based profiling into the clinical assessment of chronic wound conditions.

## Data Availability

The raw 16S rRNA sequencing data generated in this study have been deposited in the NCBI Sequence Read Archive (SRA) under BioProject accession number PRJNA1511977. The data are scheduled for public release in accordance with the NCBI release policy. Associated de-identified clinical data and supporting materials are publicly available in Figshare at https://doi.org/10.6084/m9.figshare.33198489
