# Correction: 16S rRNA sequencing and conventional culture provide complementary information in hospitalized patients with chronic lower-limb wounds

**DOI:** 10.3389/fcimb.2026.1974019

**Published:** 2026-09-14

**Authors:** Bartosz Molasy, Jarosław Rachuna, Monika Wawszczak-Kasza, Julia Dulębska, Katarzyna Kuszewska, Wioletta Adamus-Białek

**Affiliations:** 1 Institute of Medical Sciences, Jan Kochanowski University, Kielce, Poland; 2 Department of General Surgery and Coloproctology, St. Alexander Hospital, Kielce, Poland; 3 Diagnostic Laboratory, St. Alexander Hospital, Kielce, Poland

**Keywords:** 16S rRNA sequencing, chronic wounds, conventional culture, length of stay, wound healing, wound microbiome

There was a mistake in **Figures 1**–**4** as published. During the production process, the graphical contents of **Figures 1**–**4** were incorrectly assigned to the respective figure numbers. The correct assignment is as follows: **Figure 1** presents the length-of-stay analyses (≤8 days vs. >8 days); **Figure 2** presents the differential abundance analysis according to length of hospitalization (volcano plot); **Figure 3** presents the intrawound-temperature analyses (≤29 °C vs. >29 °C); and **Figure 4** presents the 12-week wound-healing analyses (No vs. Yes). The figure legends and in-text citations were correct and remain unchanged. This correction does not affect the results, statistical analyses, interpretation, or conclusions of the article. The corrected **Figures 1**–**4** appear below.

**Figure 1:**
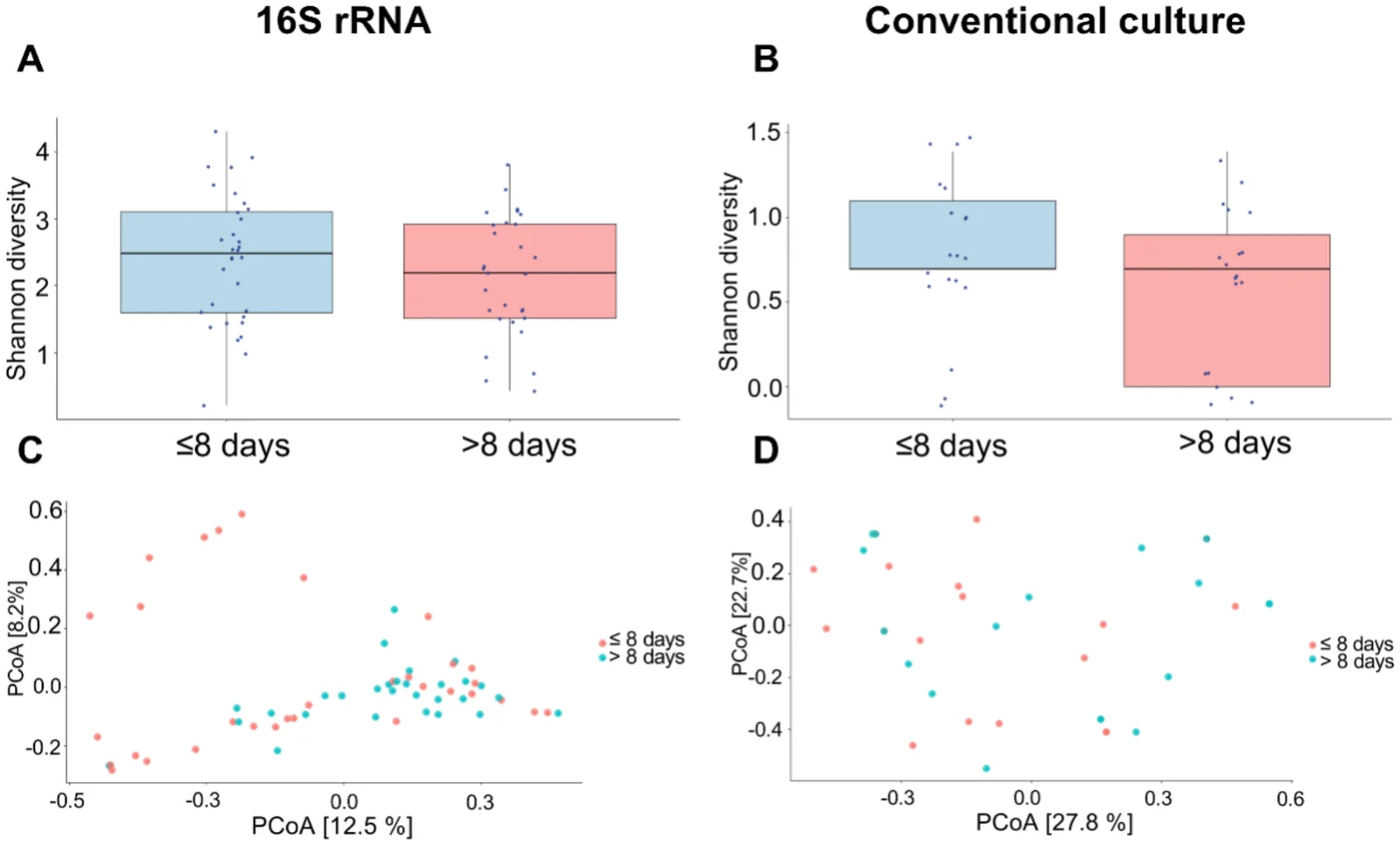
Baseline wound microbiota and subsequent length of stay in 16S rRNA sequencing and conventional culture analyses. **(A)** Alpha diversity (Shannon index) in NGS-based analysis according to length of stay (≤8 vs. >8 days). **(B)** Alpha diversity (Shannon index) in conventional culture-based analysis according to length of stay (≤8 vs. >8 days). **(C)** Principal coordinates analysis (PCoA) based on Bray–Curtis distances in NGS-based analysis. **(D)** Principal coordinates analysis (PCoA) based on Bray–Curtis distances in conventional culture-based analysis.

**Figure 2:**
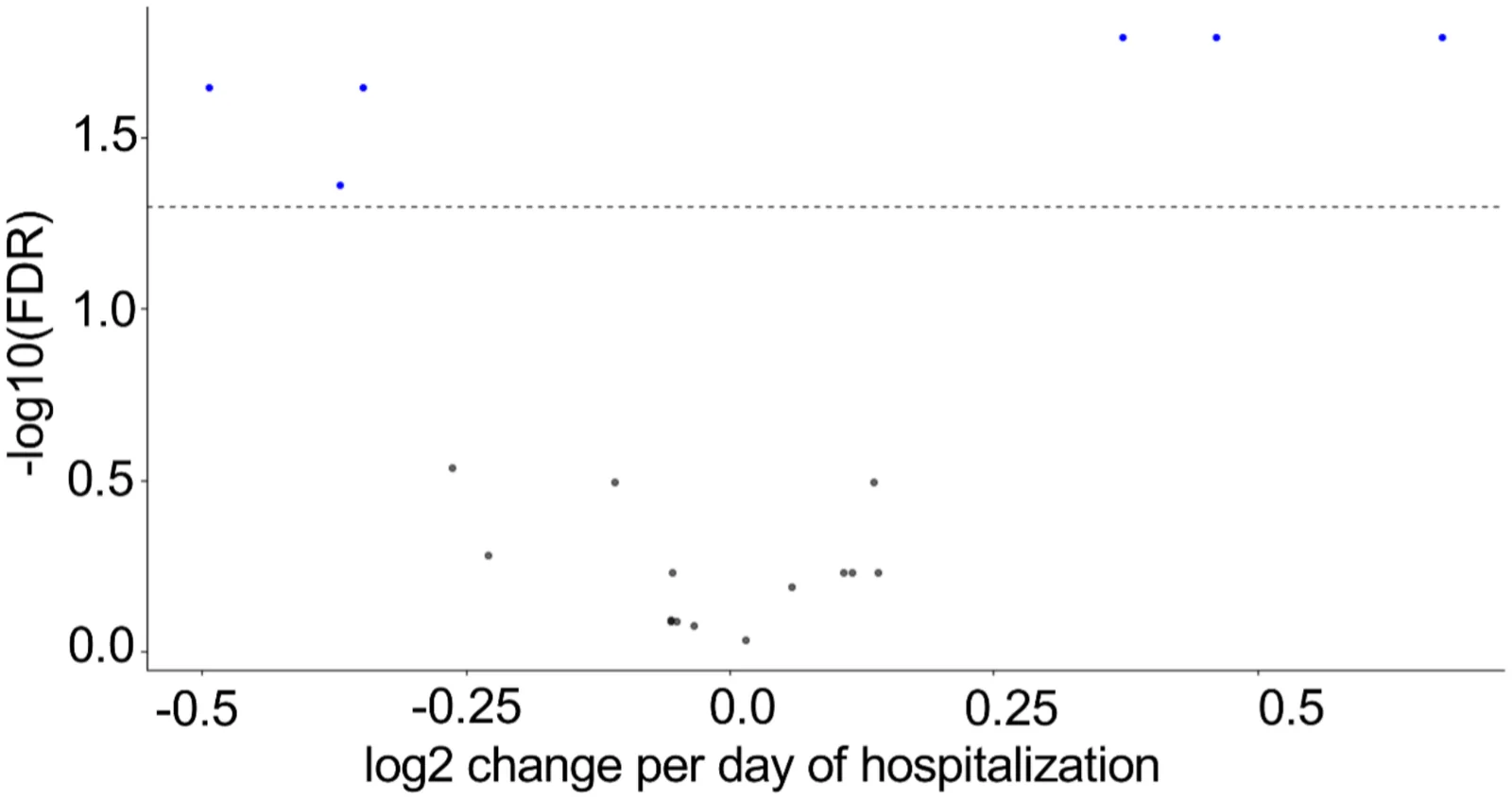
Differential abundance analysis of baseline wound microbiota in relation to subsequent length of hospital stay. Volcano plot illustrating differential abundance per day of hospitalization. Each point represents a single taxon. The dashed horizontal line indicates the significance threshold (FDR = 0.05). Blue points represent taxa with statistically significant changes, while grey points denote non-significant findings.

**Figure 3:**
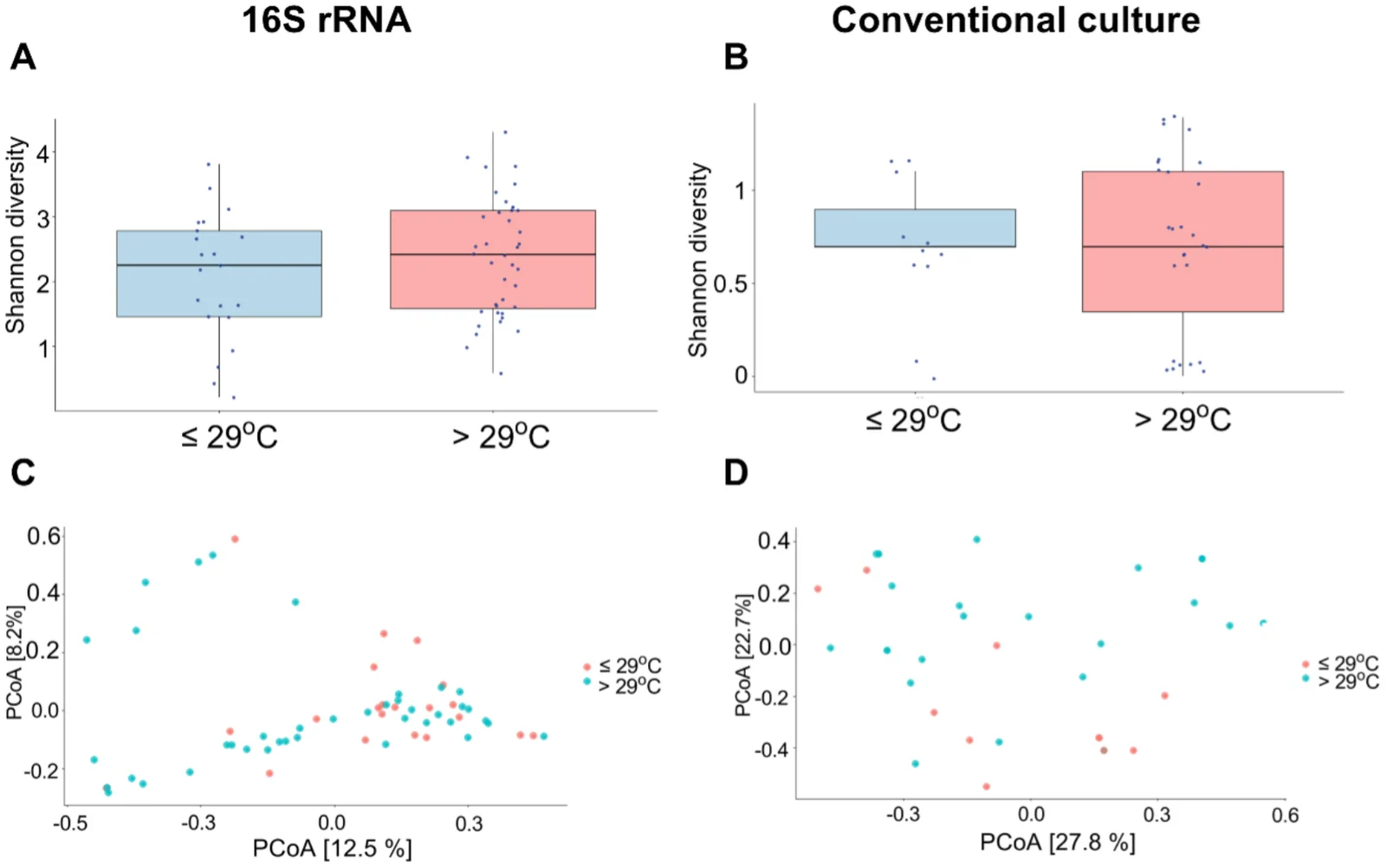
Baseline wound microbiota in relation to intrawound temperature in 16S rRNA sequencing and conventional culture analyses. **(A)** Alpha diversity (Shannon index) in NGS-based analysis according to intrawound temperature category (≤29 °C vs. >29 °C). **(B)** Alpha diversity (Shannon index) in conventional culture-based analysis according to intrawound temperature category (≤29 °C vs. ≥29 °C). **(C)** Principal coordinates analysis (PCoA) based on Bray–Curtis distances in NGS-based analysis. **(D)** Principal coordinates analysis (PCoA) based on Bray–Curtis distances in conventional culture-based analysis.

**Figure 4:**
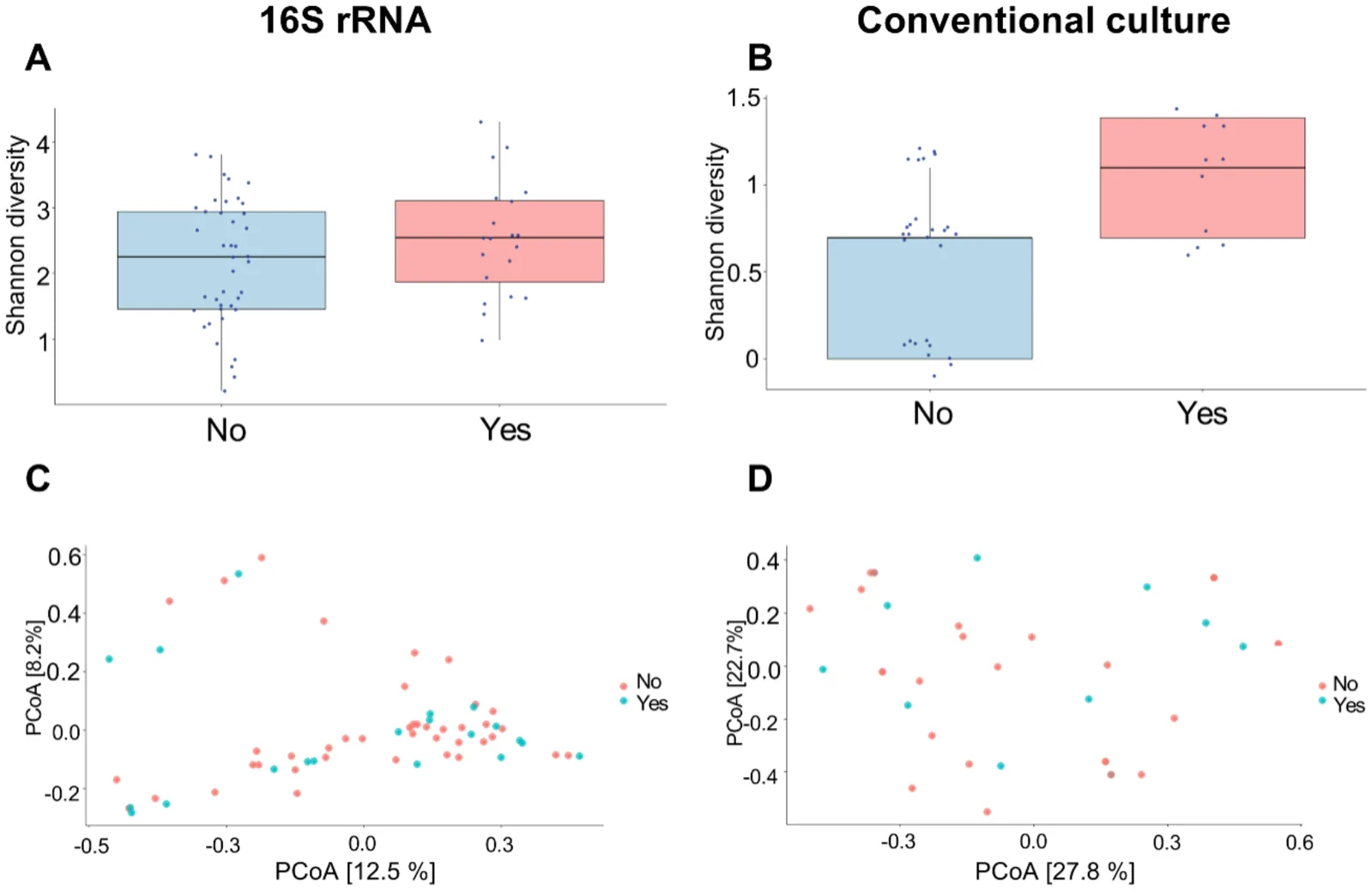
Baseline wound microbiota in relation to 12-week healing in 16S rRNA sequencing and conventional culture analyses. **(A)** Alpha diversity (Shannon index) in NGS-based analysis according to 12-week healing status. **(B)** Alpha diversity (Shannon index) in conventional culture-based analysis according to 12-week healing status. **(C)** Principal coordinates analysis (PCoA) based on Bray–Curtis distances in NGS-based analysis. **(D)** Principal coordinates analysis (PCoA) based on Bray–Curtis distances in conventional culture-based analysis.

The original version of this article has been updated.

