# Supplementary material for: 16S rRNA sequencing and conventional culture provide complementary information in hospitalized patients with chronic lower-limb wounds
Source: Front Cell Infect Microbiol. 2026 Aug 18;16:1865385. doi: 10.3389/fcimb.2026.1865385 (PMC13529466; doi:10.3389/fcimb.2026.1865385)
Supplement: Supplementary file 1 [file Table1.docx]

**Supplementary Figure S1. Phylum-level taxonomic composition of baseline wound microbiota according to hospitalization length.**

Relative abundance of dominant bacterial phyla in patients hospitalized for ≤8 versus >8 days. Proteobacteria, Firmicutes, and Bacteroidota were dominant in both groups, with modest shifts and no significant overall changes.  

**Supplementary Figure S2. Phylum-level taxonomic composition of baseline wound microbiota based on intrawound temperature category.** The dominant bacterial phyla in patients with intrawound temperatures ≤29 °C versus >29 °C remain Proteobacteria, Firmicutes, and Bacteroidota, with visual differences but no significant restructuring

**Supplementary Figure S3. Phylum-level taxonomic composition of baseline wound microbiota according to ΔT category.**

Relative abundance of dominant bacterial phyla in patients with high versus low wound-to-periwound temperature gradient (ΔT). Phylum composition was stable across ΔT groups, with Proteobacteria, Firmicutes, and Bacteroidota as the main taxa.

**Supplementary Figure S4. Phylum-level taxonomic composition of baseline wound microbiota according to 12-week healing status.**

The dominant bacterial phyla in patients with and without wound healing within 12 weeks were Proteobacteria, Firmicutes, and Bacteroidota. Both groups showed minor differences in less abundant phyla and no major changes in overall phylum profiles.
